# Spontaneous brain abscess formation: challenge of a shifting pathogen spectrum over the last 21 years – a single center experience

**DOI:** 10.1007/s00701-024-06349-8

**Published:** 2024-11-14

**Authors:** Luisa Mona Kraus, Manou Overstijns, Amir El Rahal, Simon Behringer, Klaus-Jürgen Buttler, Lukas Andereggen, Jürgen Beck, Oliver Schnell, Daniel Hornuss, Dirk Wagner, Debora Cipriani

**Affiliations:** 1 Department of Neurosurgery, Medical Center University of Freiburg, Freiburg, Germany; 2 Department of Neurosurgery, Intensive Care Unit, Medical Center University of Freiburg, Freiburg, Germany; 3 Department of Neurosurgery, Kantonsspital Aarau, Aarau, Switzerland; 4 Faculty of Medicine, University of Bern, Bern, Switzerland; 5 Department of Neurosurgery, School of Medicine, Klinikum Rechts Der Isar, Technical University Munich, Ismaningerstr. 22, 81675 Munich, Germany; 6 Department of Neurosurgery, University Hospital Erlangen, Friedrich-Alexander University Erlangen-Nürnberg, Erlangen, Germany; 7 Divison of Infectious Diseases, Department of Internal Medicine II, Faculty of Medicine, Medical Centre - University of Freiburg, University of Freiburg, Freiburg, Germany

**Keywords:** Intracerebral abscess, SAG, Odontogenic infection, Rare pathogens

## Abstract

**Background:**

Spontaneous intracerebral abscess formation is a rare condition presenting with a disabling sequela. The origin of infection can either be primary or secondary to an infection at another location. The site of primary infection - due to the proximity, often the oral cavity, the sinuses, and the orbit - determines the causative pathogens. Treatment often combines surgical and antimicrobial therapies. To determine the microbiology and respective changes and treatment outcome, we performed this retrospective monocentric cohort study of patients requiring surgical treatment of brain abscesses.

**Methods:**

Patients undergoing surgical treatment of a primary intracranial abscess between January 2000 and January 2021 in the Department of Neurosurgery, Freiburg University Hospital were included. Demographic, clinical and imaging data were extracted from patients’ medical records and databases. Treatment approaches were also analyzed, and surgical therapy and antibiotic therapy were reported. Outcome was assessed by the modified Rankin score (mRS) and was dichotomized into good (mRS 0–3) and poor (mRS 4–6) outcome.

**Results:**

We included 65 patients with spontaneous intracerebral abscess that were treated with neurosurgical intervention at our institution. Analysis of the causative pathogens showed an increasing dominance of rare pathogens such as fungi, parasites, mycobacteria and anaerobes. Outcome measured by the mRS was similar from 2005 to 2021.

**Conclusions:**

The pathogen spectrum of spontaneous intracerebral abscess at our institution is shifting with rarer pathogens being increasingly detected. This retrospective analysis highlights the need for microbiological diagnosis and of combined surgical and antibiological treatment.

## Introduction

Intracerebral abscess formation, along with epidural or subdural empyema, poses life-threatening conditions [10]. Despite advancements in modern medicine that have led to a decline in mortality rates, these remain significant at around 20% [3]. Patients with brain abscesses frequently exhibit neurological symptoms, with over half presenting with headache, often indicating intracranial hypertension [3]. Other common symptoms include nausea and confusion, while seizures are relatively rare [12].

The spectrum of organisms responsible for abscess formation varies based on the anatomical source [25]. Most spontaneous cases of intracerebral abscesses arise secondary to infections at other sites, whereas primary intracranial abscesses typically occur as postoperative complications. Interestingly, cryptogenic brain abscesses account for up to 46% of cases [25]. The most frequently detected pathogens include *Streptococcus* spp. and *Staphylococcus* spp [4, 21]; however, rarer pathogens, particularly gram-negative bacteria associated with oral flora, have been increasingly reported [23].

Key imaging techniques for diagnosing intracerebral abscesses include CT scans and MRI, focusing on T1-weighted sequences with contrast and T2-weighted diffusion sequences, such as the apparent diffusion coefficient (ADC) [9, 32]. Surgical intervention, including craniotomy and drainage by needle aspiration [20], remains a crucial component of the therapeutic approach [12]. The most commonly employed empirical antibiotic regimen consists of a combination of third-generation cephalosporins and vancomycin, with metronidazole added for anaerobic coverage [4, 7, 13]. Notably, approximately 40% of patients experience neurological sequelae following treatment [4].

## Materials and methods

### Patient data acquisition

We performed a retrospective review of adult patients with intracerebral abscess formation who were treated and operated at least one time at our institution (Department of Neurosurgery, Medical Center – University of Freiburg) from 2000 to 2021.

Eligibility criteria were.

i) confirmed spontaneous abscess formation in cranial imaging such as CT scan or MRI with contrast medium.
ii) spontaneous cause for abscess formation.
iii) surgical evacuation of intracerebral abscess either by stereotactic evacuation (needle aspiration) or open burr hole evacuation with capsule extraction.

Exclusion criteria were.

i) post-operative intracranial abscess defined as any prior history of cerebral surgery.
ii) sole epidural or subdural abscess collection.
iii) lack of follow-up.
iv) missing clinical data.
v) children (< 18 years).

Clinical data were extracted from medical archives, an emphasis was placed on the following factors.

- Micro-bacteriological analyses by culture.
- Clinical presentation prior to surgery.
- Antibiotic treatment and medical management.
- Follow-up and mRS at representation in our clinic.

Due to the design of the study, ethics board approval was obtained (22-1253-S1-retro**).**

### Statistical analyses

Descriptive statistics, including calculation of the mean and standard deviation for normally distributed and median and interquartile ranges for skewed data, were applied using GraphPad Prism (Version 10.2.3). Normal distribution was assessed graphically using boxplots and analytically using the Shapiro-Wilk test. Chi-square test was used to test for consistancy. To assess the relationship between variables Spearman’s rank correlation was used.

## Results

We conducted a retrospective analysis of patients who underwent neurosurgical treatment for intracranial abscess formation at our institution from 2000 to 2021. In total, 217 open surgeries for intracranial abscesses were performed during this period. The majority of these cases were postoperative (secondary) abscesses resulting from previous neurosurgical trepanation or other brain surgeries. Among these, 65 adult patients presented with spontaneous (primary) intracerebral abscess formation (Table 1). Diagnosis was confirmed using CT scans and/or MRI with contrast medium. Surgical treatment primarily involved open burr hole evacuation in 61 patients, while four patients were treated with needle aspiration. The median duration of intravenous antibiotic treatment was four weeks (IQR 4–6 weeks). Following surgical intervention, 58 patients were discharged alive and in improved clinical condition.

**Table 1:** Detailed cohort description

Year	Age	Sex	Pathogen	Final intravenous antibiotic treatment	Duration in weeks (+ oral)	Localization	Symptoms	Proven associated infection	Predisposing condition	mRS (FU in months)
2021	**68**	M	Fusobacterium nucleatum	vancomycin, metronidazole	4	parietal R	headache	*odontogenic*	none	0 (8)
2020	**80**	F	SAG, Parvimonas micra, Propionibacterium spp.	vancomycin, metronidazole	4 (+ 2)	right hemisphere	left hemiparesis, aphasia	**maxillary sinusitis**	history of maxillofacial surgery	3 (4)
2020	**71**	M	Nocardia paucovirorans	imipenem, cotrimoxazole	6 (+?)	parietooccipital L	confusion, speech disturbance	none	kidney transplant	3 (46)
2020	**71**	M	Nocardia abscessus	cefotaxime, cotrimoxazole	6	temporal R	none	none	B-CLL	2 (8)
2020	30*	M	Parvimonas micra, Prevotella oris	Ceftriaxone, vancomycin, metronidazole and then clindamycin per os	6	frontal L	none	orbital phlegmone, **pansinusitis**	history of tooth extraction, congenital retardation	3 (41)
2019	**24**	M	Prevotella oris	Ceftriaxone, vancomycin, metronidazole	3	frontal R	fever, headache	**pansinusiti**s	none	0 (8)
2019	**71**	M	Propriioni spp.	Piperacillin/tazobactam	4	temporal R	left hemiparesis	tympanic effusion	Temozolamid chemotherapy	4 (1)
2019	**58**	M	Staphylococcus aures	N.R	N.R.	parietooccipital R	incomplete hemianopsia	endocarditis	none	2 (12)
2019	**34**	M	Cryptococci	Amphotericin B, ceftriaxone	6	frontal R	left hemiparesis	none	HIV	2 (27)
2019	**25**	F	Staphylococcus aureus	Flucloxacillin, rifampicin	N.R.	cerebellar R	gait disturbance	none	pregnancy, Chiari malformation	0 (25)
2019	**48**	M	SAG	Penicillin	6	parietal L	right hand paresis	none	none	1 (9)
2018	**69**	F	SAG, Actinomyces odontogenicus, Fusobacterium nucleatum, Parvimonas micra, 6Staphylococcus epidermidis	Penicillin, metronidazole	6	temporal R	decreased vigilance	none	history of tooth extraction	2 (3)
2018	**72**	M	SAG	Ceftriaxone, metronidazole	6	temporo-parietal L	motor aphasia	endocarditis	none	4 (6)
2018	**28**	M	SAG	Ceftriaxone, metronidazole	6	frontal R	headache	**frontal sinusitis**	none	0 (1)
2017	**38**	F	None	none	0	occipital R	left hemianopsia	N.R	kidney transplant	3 (2)
2017	**70**	F	SAG	Penicillin, gentamycin	4 (+?)	frontal R	fever, left hemiparesis	*odontogenic*	N.R	3 (77)
*2017*	**46**	F	Candida species	AmphotericinB, anidulafungin	N.R.	temporal L	left hemiparesis, facial paralysis	none	T-LGL leucemia	4 (0.5)
2017	**35**	M	SAG, Eikenella corodens, Prevotella oris	Ceftriaxone, metronidazole	4 (+?)	temporal L	decreased vigilance, right hemiparesis, fever	*odontogenic*	Crohn’s disease	3 (2)
2016	**57**	F	SAG, Parvimonas micra, Fusibacterium spp.	Imipenem/Cilastatin, Clindamycin	4	occipital L	aphasia	*odontogenic*, encephalopathy	N.R	N.R
*2016*	**34**	M	Hyphens	Amphotericin B	N.R.	occipital R	fever, vision disturbance	none	AML	0 (1)
2016	68*	M	SAG	Penicillin	6	parieto-occipital L	global aphasia, epilepsy	*odontogenic*, **maxillary sinusitis**	N.R	4 (6)
2016	**43**	F	Nocardia farcinica	Imipenem, Cotrimoxazole	6	parietal R	decreased vigilance	none.	lung cancer	2 (10)
2016	**57**	M	SAG	Ceftriaxone	6	frontal R	hemiparesis right	none	N.R	N.R
2016	**66**	F	SAG, Parvimonas micra, Fusibacterium species	Penicillin, metronidazole	3 (+ 3)	temporal R	confusion	none	cerebral abscess 1997	3 (38)
2015	**53**	M	Streptococcus agalatiae	Penicillin, ofloxacin topical	N.R.	frontal L	right hemiparesis	Streptococcus agalatiae sepsis, endophthalmitis	alcoholism, coronary heart disease	0 (45)
2015	**61**	F	diagnosed listeria sepsis	Ampicillin	N.R.	parietal L	right leg paresis	spontaneous peritonitis	liver cirrhosis	1 (8)
2015	**84**	M	Aspergillus sp.	Voriconazole, ceftriaxone, metronidazole	4 (+ 2)	fronto-basal	headache, vision disturbance	encephalitis	B-cell lymphoma	1 (4)
2015	**82‡**	M	None	Voriconazole, piperacillin/tazobactam, amphotericin B	N.R.	central R	left leg paresis	none	B-cell non-hodgkin lymphoma	6 (2)
2014	**62‡**	M	SAG	Penicillin	N.R.	Fronto-temporal R	fall	none	alcoholism	6 (2)
2014	**80**	F	SAG	Ceftriaxone, metronidazole	6	occipital R	left hemianopsia, confusion, fall	**pansinusitis**	N.R	0 (4)
2013	**18**	M	Fungi	N.R	N.R.	frontal L	facial phlegmone	**chronic pansinusitis**	diabetes mellitus type I	0 (74)
2013	**59**	F	Staphylococcus aureus	Flucloxacillin, penicillin, metronidazole	6	temporal L, skull base involement	fever, abducens paresis	none	radiationtherapy of nasopharynx carcinoma	1 (0)
2013	**66**	F	SAG	Ceftriaxone, metronidazole	6	parieto-occipital R	left hemianopsia, and hemiparesis, dysarthria, fever	none	history of mamma carcinoma, chemotherapy	2 (19)
2013	**34**	F	SAG	Penicillin	2 (+ 4)	frontal L	motor aphasia, right hemiparesis	acute tonsillits	none	0 (3)
2012	**61**	F	Fusobacterium nucletaum	Ceftriaxone, metronidazole	4	parietal R	left hemihypesthesia	none	history of mamma carcinoma, chemotherapy	2 (17)
2012	**54**	M	None	pre-operative treatment, post-operative ceftriaxone and metronidazole	N.R.	prepontine/ suprasellar	none	E coli sepsis, cholangitis	N.R	1 (39)
2012	59*	M	Prevotella oris, Staphylococcus epidermidis	Meropenem	4	frontal R	epilepsy	**pansinusitis**, meningitis	history of tooth extraction	5 (1)
2011	**24‡**	F	Hemophilus aphrophilus	Ceftriaxone, metronidazole	4	parieto-occipital R	headache	endocarditis	Down syndrome	6 (0)
2011	**73**	F	None	Flucloxacillin, metronidazole	N.R.	retroorbital L	oculomotor paresis, vision disturbance	chronic otitis	N.R	3 (5)
2011	**44**	M	SAG	Ceftriaxone, metronidazole	6	temporo-occipital L	epilepsy, diplopia, vertigo	none	history of tooth extraction	0 (6)
2011	**37**	M	SAG	Penicillin, metronidazole	4	temporal R	dysesthesia left hand	*gingivitis*	N.R	N.R
2011	**78‡**	M	SAG	Ceftriaxone, metronidazole	N.R.	frontal R	left leg paresis	none	dilatative cardiomyopathy	6 (1)
2010	**31**	F	Streptococcus pneumoniae	Moxifloxacin	6	temporal R	decreased vigilance	otitis media, meningitis	N.R	2 (1)
2010	57*	F	SAG	Ceftriaxone, metronidazole	4	frontal R	left hemiparesis	*odontogenic*, **pansinusitis**	N.R	0 (1)
2010	**81**	F	Fusobacterium nucleatum	Cefuroxime, metronidazole	4	cerebellar R	fever, vertigo, syncope	*odontogenic*	N.R	3 (3)
2010	52*	F	SAG	Ceftriaxone, metronidazole	6	frontal R	decreased vigilance	*odontogenic*, **sphenoidal sinusitis**, meningitis	history of tooth extraction	N.R
2009	**56**	M	SAG	Ceftriaxone, metronidazole	4	parietal L	epilepsy	N.R	alcoholism	0 (1)
2009	**55**	M	None	Ceftriaxone	4	frontal R	hypesthesia left foot	N.R	N.R	3 (13)
2009	**35**	M	SAG	Ceftriaxone, metronidazole	3 (+ 3)	parietal R	facial paralysis	*odontogenic*	N.R	0 (19)
2009	**43**	F	SAG	Penicillin, metronidazole	3 (+ 3)	frontal L	headache	endocarditis	N.R	0 (4)
2008	**73**	F	Klebsiella oxytoca	Ceftriaxone, metronidazole	6	temporal L	headache	**sphenoidal sinusitis**	N.R	0 (0.5)
2008	**46‡**	F	SAG	N.R	N.R.	midbrain	epilepsy	meningitis	N.R	6 (0)
2007	**73‡**	M	Peptostreptococci, Fusobacterium, Mogibacterium, Prophyromanas gingivalis	Ceftriaxon, Ciprofloxacin, metronidazole	N.R.	cerebellar R	decreased vigilance	meningitis	liver cirrhosis	6 (0)
2007	**23**	F	none	Moxifloxacin	3	frontal L	headache	N.R	second surgery on same localization	0 (1)
2007	**23**	F	Staphylococcus aureus, Arcanobacterium haemolyticum	Flucloxacillin, metronidazole	3	frontal R	nausea, vertigo, headache	**frontal sinusitis**	N.R	0 (0)
2007	**79**	M	SAG	Ceftriaxone	2	central lesion	nausea, vertigo	*odontogenic*	history of tooth extraction	N.R
2005	**55**	M	none	Ceftriaxone, rifampicin, flucloxacillin	N.R.	multiple	decreased vigilance, fever	*odontogenic*, endocarditis	N.R	N.R
2005	**37**	F	Toxoplasma gondii	Sulfazidin, pyrimethamine	4	frontal R	facial paralysis, dysarthria	meningitis	Hepatitis B, leucopenia	N.R
2005	**54**	M	Nocardia spp.	Amikacin, imipenem, cilastin	4	parietal R	fall	*odontogenic*	CMV infection, orthotope heart transplant	1 (3)
*2002*	**31**	F	SAG	Ceftriaxon, fosfomycin	N.R.	trigonal R	headache	none	Hepatitis B and C, polytoxicomania	N.R
2002	**36‡**	M	SAG	Ceftriaxone, vancomycin	N.R.	frontal L	Epilepsy	none	Hepatitis C, polytoxicomania	6 (0)
2001	**72**	M	Proteus mirabilis, E coli, Streptococci	Ceftriaxone, metronidazole	3	parietal L	right hemiparesis, aphasia	N.R	N.R	N.R
*2001*	**43‡**	M	SAG	Ceftriaxone, vancomycin, metronidazole	N.R.	parietal R	left hemiparesis, epilepsy	meningoencephalitis	COPD	6 (1)
2000	**60**	F	SAG, Parvimonas micra	N.R	N.R.	frontal L	N.R	N.R	N.R	N.R

All 65 surgical cases of spontaneous intracranial abscess formation in adult patients at the Department of Neurosurgery, University Hospital Freiburg, from January 2000 to January 2021 were analyzed. The age ranged from 18 to 84. *Four patients* (italic style in first column) were treated by needle aspiration, 61 patients had open surgery. Of the 65 patients analyzed, 8 patients died during hospital treatment (*‡*). In four patients (*) direct association of sinusitis (bold letters) of odontogenic origin (italic letters) and subsequent intracerebral spread of the infection was found. The mRS was determined at the first available follow-up. Median time to follow-up was four months. Less than 14 days of follow-up were defined as 0

**Fig. 1:**
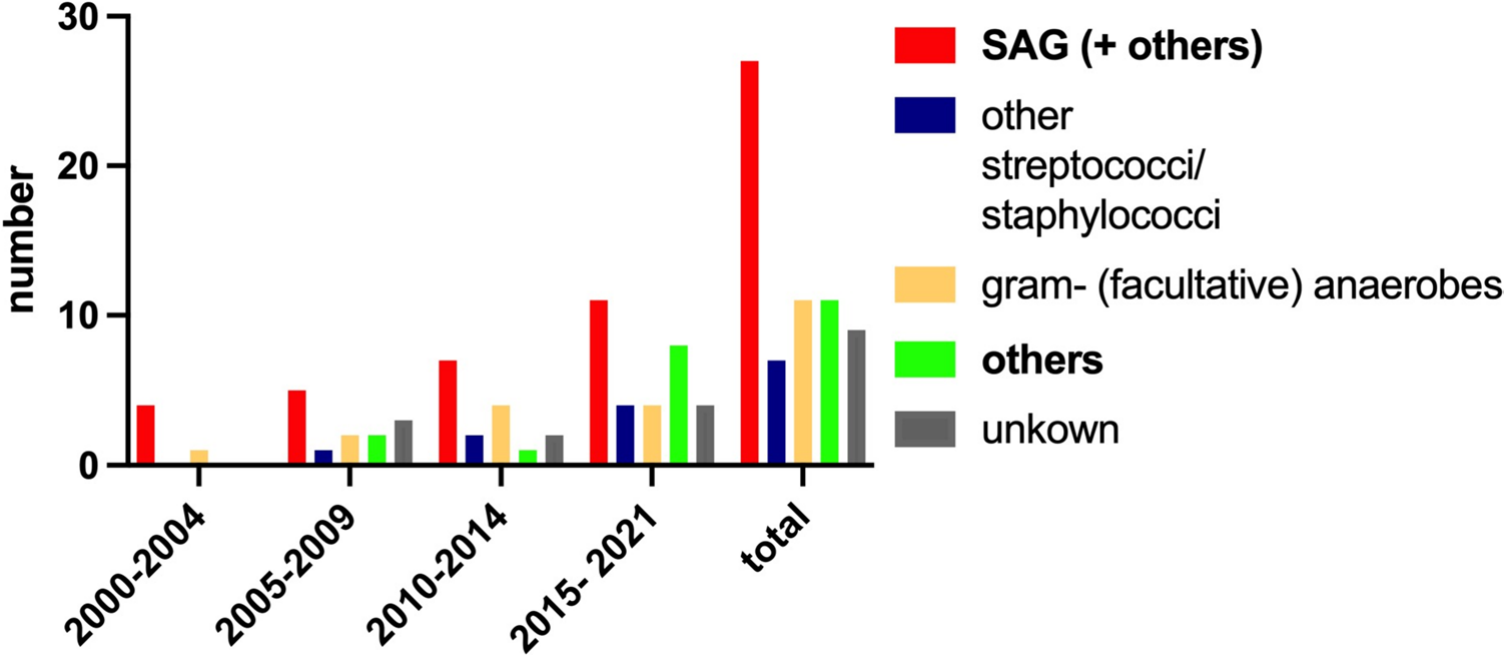
Clinical outcome at follow-up. Number of patients treated per period and clinical outcome as reported at first available follow-up. Outcome was measured by the mRS of all data available. N.R. not reported (of all patients analyzed)

Follow-up data were available for 55 patients, with a median follow-up time of four months (IQR 1–10 months). Among these, 42 patients achieved a good outcome (mRS 0–3), while five patients had a poor outcome (mRS 4–6), and eight patients died in the hospital (Fig. 1).

There was no statistically significant correlation between the duration of antibiotic treatment and clinical outcome as assessed by the modified Rankin Scale (mRS) (*p* = 0.6759). The cohort consisted of 29 females (44.61%) and 36 males (55.39%), with ages ranging from 18 to 84 years and a median age of 55 years (IQR 36–70).

Predisposing factors associated with congenital or acquired immunodeficiency included a history of malignancy and prior chemotherapy (8 patients, 12.31%), polytoxicomania (5 patients, 6.69%), history of organ transplantation (3 patients, 4.62%), liver cirrhosis (2 patients, 3.10%), and other factors such as hepatitis B, trisomy 21, diabetes mellitus, history of premature birth, congenital retardation, pregnancy, and HIV.

Associated infections included sinusitis (13 patients, 20.00%), infections of odontogenic origin (13 patients, 20.00%), meningitis or encephalitis (8 patients, 12.31%), endocarditis (4 patients, 6.15%), and endophthalmitis (1 patient, 1.54%). In five patients (6.85%), a history of odontogenic infection and simultaneous sinusitis was established.

The majority of abscesses were located in the frontal lobe (25 patients, 38.46%), followed by the parietal lobe (18 patients, 27.69%), temporal lobe (13 patients, 20.00%), and occipital lobe (5 patients, 7.69%). Other sites included the cerebellum (3 patients, 4.52%) and midbrain (2 patients, 3.08%). Only one patient was diagnosed with multiple lesions across the frontal, parietal, temporal, and occipital lobes.

**Fig. 2:**
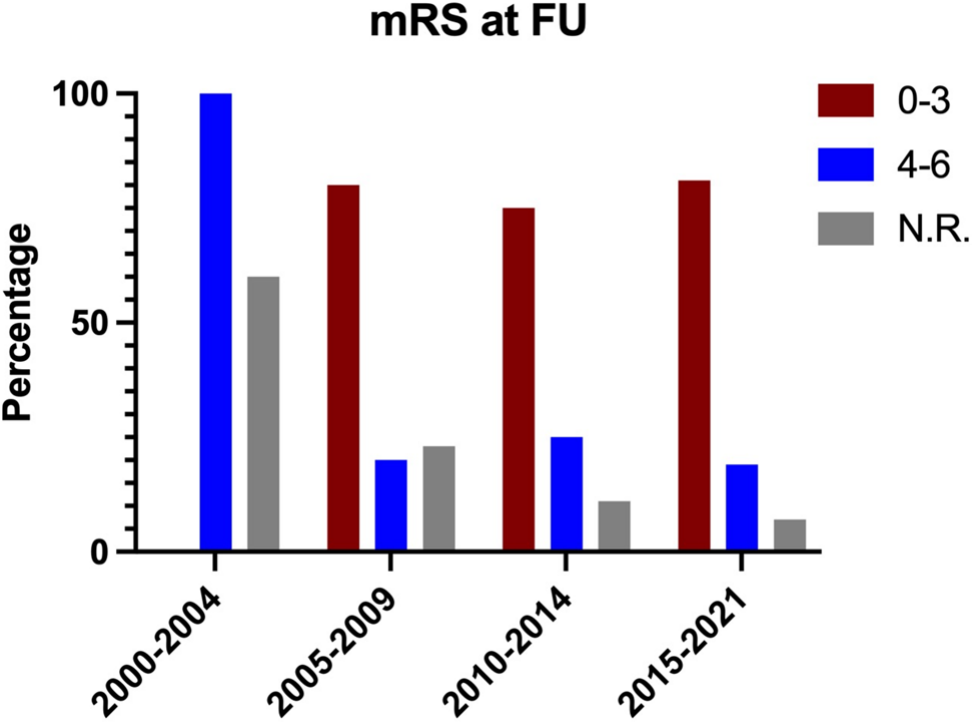
Pathogen spectrum. The pathogen spectrum of surgically treated patients with spontaneous intracerebral abscess formation at the authors’ institution was analyzed in periods of five years respectively. Groups of pathogens found in this cohort are depicted in columns: SAG and simultaneous infection with other agents (red), other gram + cocci (blue), gram- (facultative) anaerobes (light yellow), other rarer pathogens (bright green). Other pathogens are gram + rods, fungi, and parasites. The grey blue column shows the number of cases in which no pathogen could be identified

The spectrum of pathogens identified included gram-positive cocci such as *Staphylococcus aureus* and *Streptococcus* spp., gram-positive rods like *Nocardia* spp. and *Listeria* spp., gram-negative anaerobes (e.g., *Prevotella* and *Fusobacterium nucleatum*), gram-negative facultative anaerobes (e.g., *E. coli*), fungi (e.g., *Cryptococcus* spp. and *Aspergillus* spp.), and parasites (e.g., *Toxoplasma gondii*). Notably, *Streptococcus anginosus* group (SAG) was identified in 27 patients (Fig. 2), with coinfection involving SAG and gram-negative anaerobes diagnosed in six of these patients (22%), five of whom were treated in the most recent period.

An analysis of the frequency of different species of pathogens over the years revealed an increasing number of treated cases every five years (Fig. 2). Between 2000 and 2004, five cases were treated, followed by 13 cases from 2005 to 2009, 16 cases from 2010 to 2014, and 31 cases from 2015 to 2021. Throughout these periods, there were instances where no pathogen was identified: in the years 2000–2004 there were none, 2005–2009 had 3/13 (23.08%), 2010–2014 had 2/16 (12.50%), and 2015–2021 had 4/31 (12.90%). The predominant causes of infection were SAG and gram-positive bacteria across all periods. In the most recent period, the second largest group consisted of rarer pathogens classified as “others,” increasing to 25.82% (Table 2).

**Table 2:** Proportions of different pathogen species

	2000–2004	2005–2009	2010–2014	2015–2021
SAG (+ coninfetions)	80.00%	38.46%	43.75%	35.48%
Other streptococci/staphylococci	0.00%	7.69%	12.50%	12.90%
Gram-negative (facultative) anaerobes	20.00%	15.38%	25.00%	12.90%
Others	0.00%	15.38%	6.25%	**25.82%**
none found/ unkown	0.00%	23.09%	12.50%	12.90%

These rarer pathogens included *Nocardia paucivorans*, *Nocardia abscessus*, *Nocardia farcinica*, *Cryptococcus*, *Listeria*, and various fungi. Statistical analysis did not show significant changes in the proportion of SAG (*p* = 0.308) or rarer pathogens (*p* = 0.253).

## Discussion and conclusion

Epidural abscesses, subdural empyema, and brain abscesses are rare but life-threatening conditions that may arise as complications of infections such as sinusitis [12]. A brain abscess is a serious pyogenic infection affecting the cerebral parenchyma [10, 33]. Most reported cases involve brain abscess formation secondary to specific predisposing infections, with common pathogens including *Streptococcus spp.*, *Staphylococcus aureus*, and various anaerobic bacteria [4, 11]. In secondary brain abscesses, the infection can spread throufgh contiguous or hematogenous routes. Primary infections can be odontogenic or arise from sinusitis.

Our findings support the established routes of infection. Notably, one-fifth of patients in our cohort had brain abscesses accompanied by sinusitis, while another fifth presented with odontogenic infections. Additionally, 7% of patients experienced both conditions simultaneously. Interestingly, the literature often underestimates the odontogenic origin of brain abscesses [5], which contrasts with our observations [23].

In our study, we also report co-infections involving *Streptococcus anginosus group* (SAG) and gram-negative bacteria, including *Parvimonas micra* and *Prevotella oris*, both of which are typically part of the oral flora. Recent literature suggests that while anaerobic dental pathogens can be involved in sinusitis [16], their role in intracranial abscess formation is considered infrequent. Our research indicates that their pathogenicity may be underestimated.

The frontal lobe is the most common site for brain abscess formation, both in our cohort and in existing literature [14, 21, 25, 31]. This prevalence is likely due to its proximity to the sinuses and oral cavity, which facilitates contiguous spread of infection. Throughout the periods studied, SAG and gram-negative anaerobes were the predominant causes of infection, comprising 36–57% of cases. Notably, nearly one-third of all treated patients had no identifiable focus, suggesting a pattern of chronological dissemination related to the route of infection. Among odontogenic infections, SAG is frequently found in patients with primary endocarditis, pyogenic lung disease, mastoiditis, or sinusitis [2, 19, 21]. Interestingly, the majority of patients reported with severe complications from sinusitis are male [4, 18, 33].

Additionally, we wish to highlight a significant increase in the number of intracranial abscess cases requiring surgical intervention at our institution over the past two decades (Table 2). In contrast, some studies suggest a decrease in abscess cases [22, 25]. Despite the rising number of cases, our data indicate that the prognosis has not worsened; from 2015 to 2021, approximately 81% of patients had a mRS of 0–3 at follow-up, with no reported deaths. Brouwer et al. reported a significant increase in complete recovery rates when comparing outcomes from the 1960s to the 21st century [4]. Improved access to high-resolution imaging likely facilitates faster diagnoses and timely access to appropriate therapies and intensive care, which may account for the increased case numbers alongside reduced morbidity [32]. However, the median follow-up time in our cohort was only four months, and the literature describes instances of recurrent brain abscesses occurring even after long latency periods [35]. Notably, we documented a case where a patient was treated for a brain abscess 16 years prior.

Our findings indicate that infections with SAG or co-infections involving SAG and gram-negative bacteria constituted the majority of cases throughout the analyzed periods (Fig. 2). Most brain abscesses are attributed to a single pathogen, while mixed infections occur in up to 23% of cases [15, 26, 27, 30, 34]. According to Brouwer et al. (2014), fungi, parasites, and mycobacteria account for only 2% of cases [4]. While SAG remains the most common pathogen, we also observed an increasing prevalence of rarer pathogens such as *Nocardia spp.* and isolated infections with gram-negative anaerobic bacteria. This prompted an investigation into the spectrum of pathogens over the past two decades within our cohort and the broader literature. The proportion of rarer pathogens rose from 15.38% from 2005 to 2009, to 6.25% from 2010 to 2014, and increased to 25.81% from 2015 to 2021. These figures contrast with Brouwer et al.‘s meta-analysis, which reviewed the frequency of fungal, parasitic, and mycobacterial infections over approximately 70 years. It is essential to note that our study reflects a single-center experience with a limited sample size. We also want to highlight that the availability of tests and diagnostic applications has improved during recent years. Recent studies highlight the increasing role of MALDI-TOF (Matrix-Assisted Laser Desorption/Ionization Time-of-Flight) mass spectrometry and PCR (Polymerase Chain Reaction) in the diagnosis of brain abscesses, particularly when traditional methods fall short [1, 17].

Certain risk factors for acquired immunosuppression, such as prior chemotherapy, a history of organ transplantation, or alcoholism, were present in our cohort, with 25 patients (38.46%) having either congenital or acquired immunosuppression. The leading cause was a history of cancer and its treatment. Patients with HIV are at increased risk for intracranial infections [6]. The proportion of fungal brain abscesses has risen with the increased use of broad-spectrum antibiotics and immunosuppressants [28]. The number of available anti-cancer treatments has doubled from 2000 to 2015 [29]. Immunocompromised patients may be more susceptible to infections by rarer and less virulent agents. Additionally, it should be considered that pathogens normally part of the flora, such as SAG, can become more virulent in immunocompromised individuals. Intracranial infections with SAG require prolonged antibiotic treatment [8].

The emergence of a broader spectrum of pathogens necessitates a corresponding expansion in antibiotic coverage. Conversely, the increased availability of broad-spectrum antibiotics may have contributed to the development of more resistant microbial strains. Therefore, antibiotic therapy should be tailored based on microbiological results [28]. The rise in antibiotic resistance, particularly over the last two decades, presents a significant challenge in modern medicine [24]. This situation raises important questions about the extent to which we may be unintentionally driving antimicrobial resistance.

This retrospective analysis reveals a changing spectrum of pathogens in spontaneous intracerebral abscess formation. Although our findings lack statistical significance, a notable trend has emerged, highlighting the need for further studies with larger patient populations.

Ultimately, we conclude that when managing patients with spontaneous intracerebral abscesses, clinicians should consider rarer pathogens, including fungi, parasites, mycobacteria, and anaerobes associated with odontogenic infections. Moreover, our data emphasize the importance of combined surgical intervention and targeted antibiotic therapy in treating brain abscesses.

## Data Availability

No datasets were generated or analysed during the current study.

## Abbreviations

ADC: Apparent diffusion coefficient
CT: Computed tomography
FU: Follow-up
IQR: Interquartile ratio
L: Left
MALDI-TOF: Matrix-Assisted Laser Desorption/Ionization Time-of-Flight
MRI: Magnetic resonance imaging
mRS: Modified Rankin Scale
N.R.: Not reported
PCR: Polymerase chain reaction
R: Right
